# Perspective: Decolonizing postmodernist approaches to mental health discourse toward promoting epistemic justice

**DOI:** 10.3389/fpsyt.2022.980148

**Published:** 2022-10-06

**Authors:** Lia Levin

**Affiliations:** Bob Shapell School of Social Work, Tel Aviv University, Tel Aviv, Israel

**Keywords:** Foucault, postmodernism, mental health, Critical Race Theory, decolonization, epistemic justice

## Abstract

Currently, it is possible to observe a slowly (but surely) growing volume of claims seeking to disprove Foucauldian ideas about knowledge and power as overlapping basic theories of epistemic justice. Prompted by these claims, alongside adopting tenets of Critical Race Theory to address injustices inflicted upon people facing mental health challenges, I propose applying decolonizing deconstruction to Foucault's terminology, toward identifying opportunities to enhance epistemic justice, primarily in direct interventions in mental health services.

## Introduction

The rift between sociology, social philosophy, and psychiatry has been traced back to the 1970s, and is largely attributed to fundamental discrepancies between the theories, paradigms, and methodologies guiding each of these disciplines (1). One of the consequences of this split has manifested in mainstream psychiatry's broad dismissal of, or its self-preservation facing, the otherwise lively and impactful discussion surrounding post-modernity (2, 3). However, the widening preoccupation with epistemic in/justice in psychiatry and psychiatric epidemiology journals [e.g., (4–7)], signified also in the initiative to dedicate a distinct Research Topic thereto in *Frontiers*, marks an opportunity for identifying some of its notional origins and engaging in contemplation on the nuances of incorporating them into mental health discourse and practice.

## Current assumptions about the postmodernist roots of epistemic in/justice in mental health services

In recent years, it has become increasingly common to cite some of Foucault's work on postmodernism as having laid the conceptual groundwork and inspiration for the formulation of epistemic justice theory and practice (8). This appears to make a great deal of sense in light of the supposition that knowledge is essentially never neutral, and dwells in all processes involved in the construction, manufacture, institutionalization, and application of power, organically inherent to Foucault's thought and to models advocating epistemic justice (9). Both Foucauldian interpretations and approaches driven by epistemic in/justice perspectives suggest that knowledge and power are inseparable. Both point to social structures of deviance, and to the professions tasked with treating those who “deviate” from what is delimited as “normal” as agents of disciplining powers that rely on the distinction between the “truth” of scientific reason and all other forms of knowledge (10). In this vein, mental health services have often been cited as playing an impactful part in sustaining social hierarchies and perpetuating a status quo (11). Discussing Foucault employing epistemic justice terminology has also been specifically useful toward identifying injustices imposed upon those diagnosed with mental illness, and for advancing the diversification of voices shaping mental health discourses and resisting Sanism (12). Foucault himself directly addressed epistemology as a space within which status and authority are granted (and thus also withheld) (13). The apparent theoretical parallels between Foucault and ideas of epistemic justice have even prompted claims that “Michel Foucault could well be considered a theorist of epistemic justice *avant la lettre*” [Allen (14), p. 187].

## New approaches to the connection between Foucault and epistemic justice

Somewhat outside the immediate field of vision of prevailing literature, the assumption of overlap between Foucault's writing and the defining elements of epistemic justice is slowly but surely coming under criticism. This criticism takes an interesting analytical turn and applies the critical lens offered by some of the theoretical foundations of epistemic justice, namely, Critical Race Theory (15), to Foucault's philosophy itself. In doing so, some theorists point to an inconsistency between essentials of correcting epistemic injustice, namely, granting epistemic legitimacy and credibility to knowledge held by indigenous communities, sidelined social groups and experts by experience, and the seemingly heavy reliance of current debates on epistemic justice on postmodern ideas which are generated and developed primarily in privileged, Europocentric settings. The criticism drawn from this inconsistency encourages further conceptual developments of epistemic justice to keep in mind that “structuralism and post-structuralism are theoretical options born in the center of global imperialism” [Zondi (16), p. 21].

## Reconsidering postmodernist ideas for the advancement of epistemic justice

Picking up where some of said critique leaves off, two steps toward promoting epistemic justice while building on postmodern suppositions about discourse, knowledge, and power in the context of what is considered mental illness and mental health are proposed.

### Step I: Decolonizing deconstruction

The first step is applying decolonizing deconstruction to Foucault's ideas. Toward this, I refer to decolonization in its broader sense—i.e., questioning the underlying normative propositions of Western knowledge and explicitly prioritizing indigenous voices (17). In other words, decolonization as referred to in this effort is a set of assumptions regarding the need to undo and unlearn the damage caused by colonization, especially in terms of lost knowledge, silenced identities, and aggressive epistemic oppression (18). It is also a methodology that aspires to recover epistemic freedom among individuals and communities whose basic claims for self-definition have been subjected to outsiders' normative judgement and negation (19). In accordance, calls for decolonization position epistemic domination both as the result and the motivation for the colonization of the sort of knowledge that gains its justification from prejudices supporting the superiority of “white,” “Western” and “modern” viewpoints over all other knowledges or ways of knowing the world (20). Consequently, I propose that Critical Race Theory can provide helpful guidance for such decolonization and renegotiation of ideas, specifically regarding those diagnosed with mental illness. While such individuals do not fall under the classic definition of “indigenous communities” victimized by colonialization in terms of localness or heritage, their shared historical collective prosecution, systematic oppression, institutionalized discrimination, and stigmatic delegitimization arguably render them an underprivileged group, not by ethnicity or culture but by collective exclusion and shared destiny^1^. The basic assumptions underlying such an effort may thus borrow from the tenets of Critical Race Theory (24), adapted to address the mental health/illness discourse, and would constitute:

1. the idea that the dominance of Western postmodern philosophy in the analysis of discourse cannot be veiled or merited by linking it to epistemic justice;
2. the notion that Western postmodern philosophy concerning the structuring of discourse should be subjected to the same questions that it itself raises regarding the relationship between science(/theory), status, discipline, and (theoretical) authority, and the links between knowledge and power that underly such a relationship;
3. the suggestion that Western postmodern philosophy regarding the association between discourse, knowledge, and power may itself be a social construct;
4. the emphasis on storytelling and counter-storytelling, or on the role first-hand narratives can play in introducing alternative perspectives on discourses of mental health and illness; and
5. the acknowledgment that “truths” may lay beyond widely cited theories and analyses, and that Western postmodern philosophy may have not reached its status in academic and professional communities had it not capitalized on the infrastructure of knowledge dissemination and legitimation wherein its theorists enjoyed a priori advantage and access as mostly Western, mostly white, mostly educated, and mostly male.

Embracing these principles, then, entails revisiting Western notions put forward by Foucault by opening them up to alternative interpretations, underlain by the unmediated perspective of those diagnosed with mental illness.

### Step II: Focusing on testimonial epistemic justice

The second step would be redirecting some of the efforts so far inspired by Foucauldian ideas and reshaped by the decolonization and deconstruction performed in the first step toward the specific correction of testimonial epistemic injustices. Currently, literature tying Foucault's work to epistemic justice in the field of mental health more often than not addresses what can be framed as hermeneutical epistemic injustices and their relationship to sanctioned professional power [e.g., (11)]. These are the forms of epistemic injustice that lurk in the infrastructure of addressing the knowledge of members of marginalized groups, and results from an overall societal absence of skill or willingness to understand such knowledge, due to relentless exclusion of such groups from mainstream meaning-making platforms and activities. The less frequently addressed form of epistemic injustice, testimonial injustice, is the delegitimization and discrediting of an individual's account of experiences, due to bias and/or prejudice toward the social group to which s/he belongs (25). A deeper examination of the possible diverse manifestations of subjugation in micro-level encounters with mental health service-users can provide a plethora of opportunities to correct epistemic injustice, through mutual deconstruction of particular fallouts and experiences of testimonial oppression, and through employing a postmodern approach and its suggestions about the relations between discourse, knowledge, and power, as a starting point for discussion. Using Foucauldian ideas about such relationships as points of departure for dialogue with service-users and engaging in mutual examination of them, rather than keeping them in mind as underlying theoretical assumptions or treating them as secondary to the intervention process, may result in the correction of testimonial injustices so frequently prevalent in the area of mental health (26). This could begin with the foundational epistemic question “how do we know what we know about your situation, life history, challenges and strengths?” followed by, “What do we overlook or disregard in our handling of your situation, life history, challenges and strengths?” and “Why is that and what are we missing out on?”.

Presumably, focusing on testimonial epistemic justice while fostering increasing epistemic trust will enable recognition and legitimation that go beyond issues of diverse representation and subvert the structure that disciplines both those diagnosed with mental illness and those socially mandated to treat her/him.

## Discussion

Following the steps cited above could also achieve two additional goals: they could go a certain distance in grounding postmodern notions in real-life experiences of mental health services and expand theory through the entry of new, diverse voices, into the process of shaping our understanding of power structures. At the same time, they could enable the penetration of more structural theoretical understandings into the notional framework of epistemic justice, sometimes criticized for being over-individualistic and disregarding systematic factors fostering epistemic oppression, not least in the field of mental health intervention (27).

Whether interventions are articulated employing a postmodern approach and then decolonialized, guided by ideas of epistemic justice; or whether they are motivated by a desire to correct epistemic injustices and add a layer of postmodern perspectives to this effort, the status quo of mental health interventions stands to undergo transformation, as the practitioner and service-user allow themselves to engage in epistemic disobedience (28), taking various paths toward addressing knowledge and power; and contesting not only the relationship there between, but also actively diversifying their “knowledge about knowledge.” This disobedience is a key to any effort to promote justice within the complex discourse underlying many mental health and mental illness intervention settings and social constructions. Otherwise, to paraphrase American novelist Thomas Pynchon, “If they can get you to keep asking the wrong questions, they will never have to worry about answers.”

## Data availability statement

The original contributions presented in the study are included in the article/supplementary material, further inquiries can be directed to the corresponding author.

## Author contributions

The writing of this paper and the ideas that are presented in it were formulated by LL.

## Conflict of interest

The author declares that the research was conducted in the absence of any commercial or financial relationships that could be construed as a potential conflict of interest.

## Publisher's note

All claims expressed in this article are solely those of the authors and do not necessarily represent those of their affiliated organizations, or those of the publisher, the editors and the reviewers. Any product that may be evaluated in this article, or claim that may be made by its manufacturer, is not guaranteed or endorsed by the publisher.

## Footnotes

^1^Detailed discussions on the ability to apply Critical Race Theory to the analysis of disparities that are not primarily based on race without diminishing the impact of racism on mental health services are already available and inspire this suggestion (21, 22); as do recent arguments supporting such analysis as a means of observing discrimination whilst not nurturing a discourse that uses race as the ultimate explanation for social inequality (23).

## References

[1] Pilgrim D Rogers A. The troubled relationship between psychiatry and sociology. Int J Soc Psychiatry. (2005) 51:228–41. 10.1177/0020764005056987 16252791

[2] Poole R Higgo R. Postmodernism and psychiatry. Psychiatr Bull. (2009) 33:481–2. 10.1192/pb.33.12.481a

[3] Zislin J. Why postmodernism does not threaten modern psychiatry? Abstracts and questions to the problem. Neurol Bull. (2016) XLVIII:71–3.

[4] Bemme D Kirmayer LJ. Global mental health: interdisciplinary challenges for a field in motion. Transcult Psychiatry. (2020) 57:3–18. 10.1177/1363461519898035 32106797

[5] Coffey M Hannigan B Meudell A Jones M Hunt J Fitzsimmons D. Quality of life, recovery and decision-making: a mixed-methods study of mental health recovery in social care. Soc Psychiatry Psychiatr Epidemiol. (2019) 54:715–23. 10.1007/s00127-018-1635-6 30470882

[6] Crichton P Carel H Kidd IJ. Epistemic injustice in psychiatry. BJ Psych Bull. (2017) 41:65–70. 10.1192/pb.bp.115.050682 28400962 PMC5376720

[7] Miller Tate AJ. Contributory injustice in psychiatry[[Inline Image]]. J Med Ethics. (2019) 45:97–100. 10.1136/medethics-2018-104761 30337450 PMC6388905

[8] Medina J. The Epistemology of Resistance: Gender and Racial Oppression, Epistemic Injustice, and the Social Imagination. Oxford: Oxford University Press (2013). p. 352.

[9] Fricker M. Evolving concepts of epistemic injustice. In: Kidd IJ Medina J Pohlhaus G, editors. The Routledge Handbook of Epistemic Injustice. London: Routledge (2017). p. 53–60.

[10] Bracken P Fernando S Alsaraf S Creed M Double D Gilberthorpe T. Decolonising the medical curriculum: psychiatry faces particular challenges. Anthropol Med. (2021) 28:420–8. 10.1080/13648470.2021.1949892 34282672

[11] Newbigging K Ridley J. Epistemic struggles: the role of advocacy in promoting epistemic justice and rights in mental health. Soc Sci Med. (2018) 219:36–44. 10.1016/j.socscimed.2018.10.003 30359905

[12] Leblanc S Kinsella EA. Toward epistemic justice: a critically reflexive examination of ‘sanism’ and implications for knowledge generation. Stud Soc Justice. (2016) 10:59–78. 10.26522/ssj.v10i1.1324

[13] Foucault M. Aesthetics, Method, and Epistemology: Essential Works 1954–1984. New York: Penguin (2019). p. 528.

[14] Allen A. Power/knowledge/resistance: Foucault and epistemic justice. In: Kidd IJ Medina J Pohlhaus G, editors. The Routledge Handbook of Epistemic Injustice. London: Routledge (2017). p. 187–94.

[15] Dutta U Azad AK Hussain SM. Counterstorytelling as epistemic justice: decolonial community-based praxis from the Global South. Am J Community Psychol. (2022) 69:59–70. 10.1002/ajcp.12545 34363398

[16] Zondi S. Decolonising international relations and its theory: a critical conceptual meditation. Politikon. (2018) 45:16–31. 10.1080/02589346.2018.1418202

[17] Akena FA. Critical analysis of the production of western knowledge and its implications for indigenous knowledge and decolonization. J Black Stud. (2012) 43:599–619. 10.1177/0021934712440448

[18] Fanon F. The Wretched of the Earth. New York: Grove Press (1963). p. 316.

[19] Smith LT. Decolonizing Methodologies: Research and Indigenous Peoples. London: Zed (2012). p. 220.

[20] Vaditya V. Social domination and epistemic marginalization: towards methodology of the oppressed. Soc Epistemol. (2018) 32:272–85. 10.1080/02691728.2018.1444111

[21] Graham L Brown-Jeffy S Aronson R Stephens C. Critical race theory as theoretical framework and analysis tool for population health research. Crit Public Health. (2011) 21:81–93. 10.1080/09581596.2010.49317335190438

[22] Moodley R Mujtaba F Kleiman S. Critical race theory and mental health. In: Cohen BMZ, editor. Routledge International Handbook of Critical Mental Health. London: Routledge (2017). p. 79–88.

[23] Dabiri E. What White People Can Do Next: From Allyship to Coalition. New York: Penguin (2021). p. 176.

[24] Delgado R Stefancic J. Critical Race Theory: An Introduction. New York: New York University Press (2001). p. 191.

[25] Fricker M. Epistemic Injustice: Power and the Ethics of Knowing. Oxford: Oxford University Press (2007). p. 188. 10.1093/acprof:oso/9780198237907.001.0001

[26] Johnstone M. Centering social justice in mental health practice: epistemic justice and social work practice. Res Soc Work Pract. (2021) 31:634–43. doi:10.1177%2F10497315211010957

[27] Madva A. The inevitability of aiming for virtue. In: Goguen S Sherman B, editors. Overcoming Epistemic Injustice: Social and Psychological Perspectives. London: Rowman & Littlefield International (2019). p. 85–100.

[28] Mignolo WD. Epistemic disobedience: independent thought and decolonial freedom. Theory Cult Soc. (2009) 26:159–81. 10.1177/0263276409349275

